# Prevalence and predictors of problematic smart phone use among pre-varsity young people in Ibadan, Nigeria

**DOI:** 10.11604/pamj.2020.36.285.18858

**Published:** 2020-08-17

**Authors:** Folusho Mubowale Balogun, Oluwatoyin Esther Olatunde

**Affiliations:** 1 Institute of Child Health, College of Medicine, University of Ibadan, Ibadan, Nigeria,; 2 Institute of Child Health, University College Hospital, Ibadan, Nigeria

**Keywords:** Young people, problematic phone use, personality types, behavioral addiction

## Abstract

**Introduction:**

smart phone possession and use among young people is on the increase in Africa and phone addiction has been shown to be similar to substance use dependence. However, there is barely any literature focused on the effect of smart phone use on young people in Nigeria. This study looked at the prevalence of problematic smart phone use and associated factors among pre-varsity young people in Ibadan.

**Methods:**

problematic smart phone use was assessed using the 27-item mobile phone problem use scale and scored on a 5-point Likert scale. Scores 27-76 indicated low-moderate degree and scores ≥ 77 indicated moderate-severe degree of problematic smart phone use. Chi square test was used to compare problematic phone use with some selected respondents' characteristics and p was 0.05.

**Results:**

five hundred and seventy five participants were recruited, age range 14-24 years and 46.0% were males. Almost all (96.7%) of the respondents had smart phone access and 46.5% had moderate-severe problematic phone use. Young people who were males, had their parents paying for the data they use, had high extroversion, low conscientiousness and low intellect scores were likely to have problematic phone use. The identified predictors for problematic phone use were male gender (OR=1.77, 95% CI: 1.26-2.50), high extroversion (OR=1.68, 95% CI: 1.16-2.43) and low conscientiousness (OR=2.09, 95% CI: 1.41-3.09).

**Conclusion:**

there was a high rate of problematic phone use among these young people. Interventions to promote responsible smart phone use is required to counter this problem.

## Introduction

Africa has witnessed an unprecedented dramatic increase in the number of people having access to smart phones in the last ten years. About 67.0% of Africans (approximately 1.13 billion) now have smart phones [1]. Nigeria had over 154 million phone subscription in 2016 compared with 32 million in 2006 [2] and those using the internet have risen from 5.55% in 2006 to 25.67% in 2016 [3]. Many of these phones are smart phones with increasingly complex features, which makes them more appealing to young people. Phone use among young people has also witnessed exponential increase especially with the introduction of mobile learning in Nigerian schools. The variety of smart phone features and the availability of cheap internet access through service providers have made the use of smart phones more appealing to young people. Problematic phone use refers to the loss of control over phone use [4] and it has been found to affect normal human functioning and relationships. Different researchers have suggested that problematic phone use should be classified as a behavioral addiction just like excessive gambling have been so classified in the fifth edition of the Diagnostic and Statistical Manual of Mental Disorders [5]. Behavioral addictions have similarities with substance use disorders and various studies have demonstrated that individuals with behavioral addiction also exhibit craving, loss of control, withdrawal symptoms, tolerance and failure to fulfill major obligations [6,7].

They also get involved in the addicted behavior over longer time than intended, desire to reduce use, neglect important activities, continue use despite physically dangerous, psychological and social consequences [9]. Problematic smart phone use has been associated with behavioral problems [10], un-intentional injuries [11] and drug use [12] among adolescents in different settings. There was a suggestion that problematic phone use may be a combination of technological addictions (a subset of behavioral addiction) as multiple addictions can occur with phone use [13]. For example, it is possible to use the smart phone for gambling or playing of games and either of these activities have been associated with addiction [13]. It is therefore possible to have both online gambling disorder and internet addiction at the same time. This implies that problematic phone use should be taken seriously as multiple pathologies may be unnoticeably occurring concurrently. Some personality types have been implicated in behavioral addiction though the findings have been inconsistent. Extroversion and low agreeableness have been associated with problematic phone use, sexting and gambling [5,14]. The fun seeking nature of extroverts which is explained by the fact that they are naturally under-stimulated and seek external stimulation like game playing is one of the reasons that have been offered as an explanation for these problems [15].

However, Phillips *et al*. found in their study that extroversion correlated with making of calls and not game playing, which suggested that the smart phone was being used as a means of socialization by the extroverts in the study [5]. The reason for this difference is not clear. People with low agreeableness may turn to their smart phones due to the difficulties they have with interaction with other people but they were more likely to be playing games on their phone than to be using the other phone features [5]. Some studies however have reported that high agreeableness was also associated with problematic phone use [5]. Young people are more likely to experience problematic phone use because of their adventurous nature which the ever-changing innovations in smart phone features offers readily. Smart phones are portable, personal and offer immediate access to people and services. They are also able to solve almost any problems with the wide range of applications that can be installed [9]. The ability to manipulate and customize smart phones using different ringtones, screen savers and grid offers a sense of fulfillment and control. All these features are likely to make smart phones interesting for young people because it offers them the personal autonomy, identity and prestige they desire [16] which aligns naturally with their social development. Smart phones are also tools of socialization for young people and it can be used to establish and maintain social relationships.

This is aided by the different social media applications which keep increasing in number. However, because of their fun seeking nature and their developing prefrontal cortex (which is responsible for making judgement) [17], they are more likely to be prone to problematic phone use. This has been demonstrated by different studies with prevalence of problematic smart phone use ranging from 2.4% to 21.3% among young people in the literature [12,18,19]. However, the literature regarding problematic phone use among young people in sub-Saharan Africa and Nigeria is scarce despite the increase in phone possession and use among them. This study is a sub-set of a larger study which looked at the prevalence and predictors of sexting among post-secondary school young people in Ibadan in Nigeria. This particular segment of the study set out to describe phone access and phone use among pre-varsity young persons in Ibadan, Nigeria and to determine the prevalence of problematic phone use, its associated factors and predictors among them. It is important to know the prevalence of problematic phone use among this important age group as there may be peculiarities which are yet to be documented particularly in our environment.

## Methods

**Study design:** this was a descriptive cross-sectional study design which used self-administered questionnaire for data collection.

**Study area:** the study was conducted in Ibadan metropolis which is made up of five local government areas in Oyo state. The state ministry of education is responsible for the administration of both the advanced education centers and the pre-varsity preparatory centers. There were six registered advanced education centers and thirty registered pre-varsity preparatory centers at the time of the study.

**Study participants:** the participants were young people aged 10 to 24 years who were attending advanced education centers and pre-varsity preparatory centers. They were purposely selected as they were more likely to own and use phones.

**Sample size calculation and sampling:** this was reported in an earlier publication which reported the findings from the main study that investigated the prevalence and predictors of sexting from the study population [14].

**Data collection procedure:** selected participants completed a self-administered semi structured questionnaire with questions regarding the socio-demographic characteristics, phone access and phone use. There were also questions investigating personality traits using the 50-item set of International Personality Item Pool (IPIP) Big-Five Factor Markers [20] and problematic smart phone using Mobile Phone Problematic Use scale (MPPUS), the 27-item instrument designed by Bianchi and Phillips [15].

**Ethical consideration:** all procedures performed in studies involving human participants were in accordance with the ethical standards of the Oyo State Ethics Review Committee and in accordance with the 1964 Helsinki declaration and its later amendments. Ethical approval was obtained from the Oyo State Ethics Review Committee of the State Ministry of Health (Reference number: AD 13/479/938), in Ibadan. Permission to conduct the study was first obtained from the heads of schools, with written informed consent obtained from students aged 18-24 years. Students less than 18 years provided assent. Voluntariness, privacy, confidentiality and anonymity were strictly maintained.

**Data analysis:** data was analyzed using Statistical Package for Social Sciences (SPSS) version 22. The socio-economic class of the study participants was computed using the socio-economic index scores of Oyedeji in which socio-economic index scores were awarded to each of the study participant based on the occupations and educational attainment of the parents. In this classification, the highest socio-economic class is I while V is the lowest [21]. The 50-item set of International Personality Item Pool (IPIP) Big-Five Factor Markers was used to measure the personality traits of the respondents [20]. The IPIP has 10 questions statements each for five different personality traits. For example, some of the statements used to measure extroversion were: I am the life of the party; Feel comfortable around people; Don´t talk a lot. Also, examples of questions used to measure conscientiousness were: I am always prepared; Leave my belongings around; Pay attention to details. Each statement was scored on a 5-point Likert scale. When the overall score for each personality trait is less than the mean score of the trait, it indicated a low score for that personality trait, and an overall score at or greater than the mean score indicated a high trait score. Each question was scored on a 5-point Likert scale and mean scores were determined for each trait among the study participants. Those who score less than the mean score for any trait were categorized as low score for that trait. The Chronbach alpha for this instrument was 0.60 in this study group. Each item was scored on a 5-point Likert scale. When the overall score for each personality trait is less than the mean score of the trait, it indicated a low score for that personality trait, and an overall score greater than the mean score indicated a high trait score.

**Validation and test of reliability of mobile phone problematic use scale:** following content validation, the item “I have received phone bills I could not afford to pay” was rephrased as “I have used money that was more than intended in my budget to buy airtime/data on my phone” because prepaid airtime/data procurement is the norm in the study area. MPPUS have been reported to be reliable tool for self-reported phone use [15] but its reliability is yet to be tested among Nigerian young people. The 27 items provide information about mobile phone addiction, disruption of normal day to day relationships and functioning and withdrawal symptoms. The items were scored on 10-point Likert scale. The Chronbach alpha for MPPUS was 0.88 for the sample, the scale mean was 75.29 and standard deviation was 18.16. The mean score and standard deviation for each parameter is shown in Table 1.

**Table 1:** parameters and reliability of the smart phone problem use scale

Problematic phone use parameters	Item M	Item SD	Corrected item-total correlation	Alpha if item deleted
I can never spend enough time on my smart phone	2.91	1.21	0.02	0.88
I have used my smart phone to make myself feel better when I was feeling down	3.87	1.12	0.27	0.88
I find myself occupied on phone when I should be doing other things and it causes problems	3.04	1.29	0.45	0.87
All my friends own phone	3.84	1.13	0.18	0.88
I have tried to hide from others how much time I spent on my smart phone	2.40	1.16	0.46	0.87
I lose sleep due to the time I spend on my smart phone	2.69	1.42	0.59	0.87
I have used money that was more than intended in my budget to buy airtime/data on my phone	2.08	1.20	0.37	0.87
When out of range for some time, I become preoccupied with the thought of missing a call	2.67	1.35	0.44	0.87
Sometimes, when I am on the smart phone and I am doing other things, I get carried away with the conversation and I don´t pay attention to what I am doing	3.08	1.35	0.55	0.87
The time I spend on my smart phone has increased over the past 12 months	2.75	1.29	0.52	0.87
I have attempted to spend less time on my smart phone but I am unable to	2.71	1.25	0.56	0.87
I have used my smart phone to talk to others when I was feeling isolated	3.49	1.22	0.26	0.88
I find it difficult to switch off my smart phone	2.56	1.38	0.55	0.87
I feel anxious if I have not checked for messages or switched on my smart phone for sometime	2.90	1.37	0.56	0.87
I have frequent dreams about the smart phone	1.96	1.17	0.45	0.87
My friends and family complain about my use of the phone	2.70	1.36	0.59	0.87
If I do not have a smart phone, my friends would find it hard to get in touch with me	3.50	1.30	0.34	0.87
My productivity has decreased as a direct result of the time I spend on the phone	2.47	1.25	0.55	0.87
I have aches and pains that are associated with my smart phone use	2.38	2.48	0.30	0.88
I find myself engaged on phone for longer periods of time than I intended	2.85	1.34	0.59	0.87
There are times when I would rather use the phone than deal with other pressing issues	2.89	1.85	0.43	0.87
I am often late for appointments because I´m engaged on the phone when I shouldn´t be	2.12	1.46	0.33	0.87
I become irritable if I have to switch off my phone for meetings, dinners or at movies	2.43	1.45	0.49	0.87
I have been told that I spend too much time on my smart phone	2.84	1.35	0.56	0.87
More than once I have been in trouble because my phone has gone off during meeting	2.39	1.27	0.50	0.87
My friends don´t like it when my phone is switched off	3.12	1.30	0.35	0.87
I feel lost without my smart phone	2.65	1.40	0.51	0.87

M: Mean SD: Standard deviation

**Categorization of the MPPUS scores:** the MPPUS score for each student was categorized first into three categories as indicated by Smetaniuk in 2013 (low-moderate degree of concern: 27-76; moderate-high degree of concern: 77-126; high-severe degree of concern: 127-231 and above) [7] and then into two categories by collapsing moderate-high degree and high-severe degree into one group named moderate-severe degree (77-231 and above). Total score ≤ 76 indicated low-moderate degree and score ≥ 77 indicated moderate-severe degree of problematic smart phone use. For this study, problematic mobile phone use was defined as scores from 77 to 231. Data was analyzed using descriptive statistics and Chi square test was used to compare some selected sociodemographic characteristics with problematic phone use parameters. Multinomial logistic regression was used to obtain the predictors of problematic smart phone use and the level of significance was set at 5%.

## Results

**Sociodemographic characteristics of respondents:** the mean age of the study participants was 17.4±2.0 years, majority (472, 86.0%) were adolescents (aged 10 to 19 years) and the remaining were aged 20 to 24 years. There were 304 (54.0%) females and 454 (78.9%) were living with both parents. The socioeconomic class distribution was such that 157 (27.8%), 328 (58.2%), 70 (12.4%) and 9 (1.6%) belonged to socio-economic class I, II, II and IV respectively. There was no respondent belonging to socioeconomic class V. Most (487, 85.9%) were from monogamous family and 501 (87.6%) had parents who were married.

**Phone access and phone use of the respondents:** almost all the respondents (96.7%) had access to cellphones and these were mainly personal phones with smart phone features. Data used for the phones were mainly from the parents of the young people and those that they purchased themselves. These phones are usually used to surf the internet, to make calls and send text messages. Table 2 has other details about the study participants' phone access and use.

**Table 2:** phone access and use of respondents

Variable	Frequency	Percentage (%)
**Phone access**		
Have access to a phone	556	96.7
Have a personal phone	552	96.3
**Type of phone**		
Smart phone	476	82.8
Not a smart phone	69	12.1
**Person paying for data used**		
Self	378	66.2
Parents	244	42.9
Friends	38	6.7
Others	35	6.2
**Phone use**		
Surf the internet	375	65.7
Call friends	353	61.8
Send text	310	54.3
Send pictures/videos	214	37.5
Take pictures/video	161	28.2
**Frequency of using phone to access social network sites**		
Never	50	9.2
>1/month	32	5.9
1-2 times/month	43	7.9
1-2 times/week	203	37.5
Daily	213	39.4

**Problematic phone use assessment:** two hundred and sixty five (46.1%) respondents had moderate to high problematic phone use which was significantly associated with the male gender and parents paying for data. More respondents with moderate to severe problematic phone use were from polygamous families but this was not statistically significant. Table 3 shows the other association of problematic phone use with selected characteristics of the study participants.

**Table 3:** association between selected respondents characteristics and problematic phone use

Parameters	Problematic phone use score		p value
	Low to moderate	Moderate to severe	
**Sex**			
Female	140(54.1%)	119(45.9)	<0.01
Male	120(40.1%)	180(60.0)	
**Age**			
≤19 years	253(53.9)	216(46.1)	0.65
>19 years	52(51.5)	49(48.5)	
**Socioeconomic class**			
Low	5(55.6)	4(44.4)	0.91*
High	296(53.7)	255(46.3)	
**Family type**			
Monogamous	267(55.3)	216(44.7)	0.06
Polygamous	35(43.8)	45(56.3)	
**Parents marital status**			
Married	269(54.0)	229(46.0)	0.52
Others	36(50.0)	36(50.0)	
**Who participant lives with**			
Both parents	250(55.1)	204(44.9)	0.14
Others	55(47.4)	61(52.6)	
**Cell phone access**			
Yes	292(52.9)	260(47.1)	0.11
No	13(72.2)	5(27.8)	
**Parents pay for participant’s data**			
Yes	100(41.2)	143(58.8)	0.03
No	163(50.6)	159(49.4)	
**Extraversion score**			
High	144(47.2)	161(52.8)	0.02
Low	157(60.4)	103()39.6	
**Agreeableness score**			
High	156(57.0)	115(42.4)	0.07
Low	149(49.8)	150(50.2)	
**Conscientiousness score**			
High	175(61.8)	108(38.2)	<0.01
Low	130(45.5)	156(54.5)	
**Emotion score**			
High	181(52.8)	162(47.2)	0.54
Low	123(55.4)	99(44.6)	
**Intellect score**			
High	187(58.8)	131(41.2)	0.01
Low	117(47.0)	132(53.0)	

**Factors associated with problematic phone use and predictors:** predictors of problematic phone use as shown in Table 4 include the male gender, high extroversion and low conscientiousness.

**Table 4:** predictors of problematic smart phone use

Characteristics	Unadjusted estimates		p value	Adjusted estimates		p value
	OR	95% CI		OR	95% CI	
**Sex**						
Female	1	-		1		<0.01
Male	1.81	1.29, 2.54		1.77	1.26, 2.50	
**Parents pay for participant´ data**						
No	1	-	0.26	1	-	0.38
Yes	1.47	1.05, 2.05		1.18	0.82, 1.69	
**Extraversion score**						
Low	1	-	<0.01	1	-	0.01
High	1.70	1.18, 2.43		1.68	1.16, 2.43	
**Conscientiousness score**						
High	1	-	<0.01	1	-	<0.01
Low	1.86	1.28, 2.71		2.09	1.41, 3.09	
**Intellect score**						
High	1	-	0.29	1	-	0.50
Low	1.23	0.84, 1.8		1.14	0.77, 1.70	

aCorrection done for age, family type, phone access and socioeconomic class

## Discussion

There is a large number of young people who have moderate to severe problematic phone use in this study population and this prevalence is higher than what have been reported in the literature both in sub-Saharan Africa and beyond [7,12,18,22]. This should be of great concern as it is likely the problem is yet to be recognized in the study population. This implies that the immediate and long-term consequences of problematic phone use is likely to be enormous in Nigeria which has the highest number of adolescents in Africa and by extension, could have the largest cohort of African young people. This justifies taking a closer look at the problem. The phone possession rate and percentage of those with smart phone are also higher than what is reported for the country [2,23]. Phone ownership and access in Africa have been reported to be more among people who are young, English speaking and educated [23]. The study population have these characteristics as they were young students who were pursuing higher learning to proceed to tertiary institutions. English is also the official language in Nigeria and it is used in teaching in the schools. Almost all the phones have smart phone features which broadens the capability of the phones as internet use makes it possible to download different applications which can be used for different purposes. It has been shown that literacy is associated with the use of smart phones because literate people are able to navigate the web easily compared with those who are not literate.

The study population were mainly in the higher socioeconomic class and this can explain their ability to afford the phones and buy airtime and data. The lowest rung of socioeconomic class are less likely to pursue higher learning and this may explain their complete absence in this study. The commonest activity that the smart phones were being used for is surfing of the internet. This is likely to expose the respondents to different online activities which may be beneficial or deleterious to them. The proportion that visit social network sites in the present study is above the average of 35.0% that was reported for Nigeria [23]. The attributes responsible for increase smart phone ownership rate that was discussed earlier may also explain this high access to social network sites. Young people are also more likely to be interested in socializing and this can be the attraction for social networking sites. This is likely to predispose them to other problems of such sites like sexting [14] and cyber-bullying [24] which have been reported in the study environment. These are different problems that have been associated with smart phone use and reiterate the reason why phone use among young people in Nigeria need to be scrutinized. Clearly more than a third of the young people demonstrated features in keeping with problematic phone use.

Although this study did not set out to pathologize phone use, the constructs of MPPUS included questions on addiction (which have been similarly listed in behavioral addictions) and other things which raises concern about the pattern of phone use in the study group making it is important to discuss them. Mood modification, conflict (both with self and others) and preoccupation can negatively affect social interaction which is required for normal development and functioning. It can also affect the process of learning which the respondents are expected to be actively involved in and this can affect the productivity of these young people in adulthood. This study group had a significant number of respondents with inadequate sleep due to phone use. Adequate sleep has been associated with better cognitive and mental functioning as well as general well being and inadequate sleep has been reported among adolescents in the study area [25]. Poor sleep quality has also been reported to be associated with problematic smart phone use in a study among Turkish university students [26]. This can also affect learning and social interactions. It is interesting to note that longer hours of sleep was protective against problematic phone use among young adolescents in Italy [19]. Some respondents have made some efforts at controlling the amount of time spent on phone but have failed. This is likely to lead to frustration and triggering of other problems.

The male gender was not only associated with problematic smart phone use in this study but was a strong predictor of the problem after controlling for the effects of possible confounders like age, family type, phone access and socioeconomic class. This may be as a result of the extra time at the disposal of the male gender as females of the same age culturally are more involved in house chores aside the demand of their academics and social relationships and are more generally held responsible for their actions by their parents compared to the boys. This is in contrast with the reports from similar studies where the female gender was associated with problematic smart phone use [10,12,27,28]. Although age was not associated with problematic phone use in this study, there have been inconsistent reports about the association between problematic phone use and age in earlier studies [27,29] and these differences cannot be explained by the differences in instruments used to assess problematic phone use as they were similar. Further research is required to clearly define the relationship between age and problematic phone use if there is any. This is the same for the association between socioeconomic class and problematic phone use. The personality types that predicted problematic phone use were high extroversion, low agreeableness and low intellect. High extroversion consistently predicted problematic phone use in this study, similar to earlier reports [7,30]. This personality trait have been associated with impulsive nature and addictive behaviors which is likely to be due to their sensation seeking nature and this has been linked with risk taking [5].

Low conscientiousness was also a predictor of problematic phone use in this study which is in contrast to the finding by Takao *et al*. in India [30]. This personality trait is associated with difficulty in achieving goals [5] and they may find solace in the use of their smart phones leading to addiction. Low intellect also predicted problematic phone use but it was no longer a significant predictor after controlling for age, family type, phone access and socioeconomic class.

## Conclusion

In conclusion, young people in this study had a somewhat high level of problematic phone use and it was associated with the male gender, extraversion and low conscientiousness personalities. Even though they used their smart phones for apparently normal day to day activities, there is a need for caution as problematic phone use can disrupt normal functioning of these young people who are at an important stage of development. This calls for efforts at different levels to promote responsible smart phone use among these young people. This study has some shortcomings which should be acknowledged. Firstly, the study was conducted among young people who are preparing for higher education and they are from the high and middle socioeconomic class. This can limit the application of the findings in this study to other population of less educated young people. For example, problematic phone use have been reported to be higher among adults who were less educated [31]. Secondly, the study is also cross sectional and so, causality cannot be ascertained. Lastly, the respondents reported their behaviors with regard to phone use which may not be the true situation as reported phone use have been shown not to be the same as actual use sometimes. However, this study highlighted the problems of problematic phone use among the young people and its predictors and its finding may be useful in creating awareness about the problem and designing impactful interventions to address it. To successfully address this type of problem, the involvement of the people in the immediate environment of the young people and provision of some specialized skills is required. Tailoring these interventions to the sociocultural settings in the study area will also ensure that problematic phone use is addressed in a holistic way.

### What is known about this topic

- There is a large variation in the pattern of young people's smart phone use;
- Young people have the tendency to have problematic smart phone use in Asian and western countries;
- There are factors already associated with problematic smart phone use among young people but these have not been consistent in different studies.

### What this study adds

- There is a relatively a high prevalence of problematic smart phone use among these young people;
- Problematic smart phone use was predicted by the male gender, high extroversion and low conscientiousness personalities among this Nigerian young people.
